# Genomic and Molecular Associations with Preoperative Immune Checkpoint Inhibition in Patients with Stage III Clear Cell Renal Cell Carcinoma

**DOI:** 10.3390/cancers18020312

**Published:** 2026-01-20

**Authors:** Wesley H. Chou, Lucy Lawrence, Emma Neham, Shreeram Akilesh, Amy E. Moran, Christopher L. Corless, Lisa Langmesser, Beyza Cengiz, Kazumi Eckenstein, Jen-Jane Liu, Sudhir Isharwal, Christopher L. Amling, Marshall C. Strother, Nicholas H. Chakiryan, George V. Thomas

**Affiliations:** 1 Department of Urology, Oregon Health & Science University, Portland, OR 97239, USA; chouw@ohsu.edu (W.H.C.); lawrenlu@ohsu.edu (L.L.); langmess@ohsu.edu (L.L.); jenj@ohsu.edu (J.-J.L.); isharwal@ohsu.edu (S.I.); amling@ohsu.edu (C.L.A.); strothem@ohsu.edu (M.C.S.); chakirya@ohsu.edu (N.H.C.); 2 Department of Cell, Developmental and Cancer Biology, Oregon Health & Science University, Portland, OR 97239, USA; emmajuneneham@icloud.com (E.N.); moranam@ohsu.edu (A.E.M.); 3 Knight Cancer Institute, Oregon Health & Science University, Portland, OR 97239, USA; corlessc@ohsu.edu (C.L.C.); cengiz@ohsu.edu (B.C.); kazumi@ohsu.edu (K.E.); 4 Department of Laboratory Medicine and Pathology, University of Washington, Seattle, WA 98195, USA; shreeram@uw.edu; 5 Department of Pathology and Laboratory Medicine, Oregon Health & Science University, Portland, OR 97239, USA; 6 Department of Urology, Portland VA Medical Center, Portland, OR 97239, USA

**Keywords:** renal cell carcinoma, transcriptomics, proteomics, immune checkpoint inhibition

## Abstract

**Background and Objective**: Patients with stage III clear cell renal cell carcinoma (ccRCC) have a high risk for disease recurrence post-nephrectomy. To mitigate overtreatment, there is a pressing need to determine who benefits from immune checkpoint inhibition (ICI) around the time of surgical resection. We performed digital spatial analysis of both gene and protein expression in stage III ccRCC tumors, some of which had preoperative ICI exposure. **Methods**: Nephrectomy specimens from stage III ccRCC patients were analyzed using the Nanostring GeoMx Digital Spatial Profiler. Differential expression analysis was performed and validated using NCT02210117 trial data to identify genes associated with both ICI and clinical response. A gene score was then generated to predict overall survival in patients from The Cancer Genome Atlas (TCGA). **Key Findings and Limitations**: In a small cohort of 19 patients, RNA expression significantly differed based on preoperative ICI exposure and recurrence status—CD8+ effector and central-memory T-cell signatures were less prevalent in the treatment-naïve with recurrence group. Three out of four patients with preoperative immune checkpoint inhibition recurred. External validation yielded a four-gene set (*GZMK*, *GZMA*, *ITGAL*, and *IL7R*), where higher expression levels predicted better overall survival in the TCGA cohort (*p* = 0.005). **Conclusions and Clinical Implications**: Preoperative ICI favorably altered the tumor microenvironment to resemble that of treatment-naïve patients without recurrence but did not translate to improved survival. Upon external validation, the genes *GZMK*, *GZMA*, *ITGAL*, and *IL7R* were modifiable with ICI and associated with improved overall survival. Further investigation is needed to assess if patients with low baseline expression of these genes may benefit from ICI around the time of surgery.

## Simple Summary

Existing clinical data regarding immune checkpoint inhibition around the time of surgery in patients with stage III clear cell RCC are conflicting. To better understand the effects of immune checkpoint inhibition on the tumor microenvironment, we performed differential expression analysis of proteins and RNA in surgical specimens from patients with stage III clear cell RCCs. We found that while preoperative immune checkpoint inhibition altered the tumor microenvironment to resemble that of treatment-naïve patients without recurrence, these changes did not necessarily translate to improved oncologic outcomes. Specifically, prevalence of CD8+ T effector and central memory cells appeared lower in the treatment-naïve patients who had recurrence. Upon external validation, we identified the genes *GZMK*, *GZMA*, *ITGAL*, and *IL7R* as being associated with both exposure to immune checkpoint inhibition and improved survival. When expression of these four genes was assessed in cohorts from The Cancer Genome Atlas (TCGA), higher expression levels were associated with better overall survival. These genes should be further investigated as potential predictors for benefit from immune checkpoint blockade.

## 1. Introduction

Patients with stage III clear cell renal cell carcinoma (ccRCC) are at a high risk of recurrence after surgical resection—historically, this has varied between 30 and 50% [1]. There have been substantial efforts to develop new treatment strategies incorporating immune checkpoint inhibitors (ICIs) to minimize the risk of recurrence after surgery [2]. The sole positive study in this domain has been KEYNOTE-564, a randomized controlled trial that found that adjuvant pembrolizumab improved disease-free survival versus placebo in ccRCC patients at increased risk for recurrence (hazard ratio [HR]: 0.72, 95% confidence interval [CI]: 0.59–0.87) [3]. Based on these results, the current National Comprehensive Cancer Network (NCCN) kidney cancer guidelines recommend adjuvant pembrolizumab versus surveillance for surgically resected stage III ccRCCs [4]. In contrast, results from the PROSPER trial failed to show clinical benefit when preoperative nivolumab was used in a similar cohort (HR: 0.94, 95% CI: 0.74–1.21) [5].

There remains a pressing need to determine which stage III ccRCC patients benefit from ICI therapy around the time of surgery. A better understanding of this would avoid treatment for patients who can be cured with surgery alone and reserve treatment for patients who stand to derive the most benefit. Additionally, there is still much to be described regarding the effect of ICIs on the tumor immune microenvironment of stage III ccRCC cancers and how these changes may be associated with clinical outcomes. We performed a multimodal digital spatial analysis of gene and protein expression in tumors from patients with stage III ccRCC, some of whom had been exposed to preoperative ICI—with additional refinement and validation using several independent cohorts.

## 2. Methods

### 2.1. Tumor Samples

Stage III ccRCC patients who had undergone nephrectomy for primary tumors between 2016 and 2021 were identified from our institution’s prospective biorepository (*n* = 19). All patients consented to data and specimen collection and subsequent analysis. Approval was obtained from our institution’s local institutional review board (IRB). The approval number for this protocol was Oregon Health & Science University- (OHSU) IRB #4918.

Patients had ≥48 months of recurrence-free postoperative follow-up or biopsy-proven clinical recurrence within that timeframe. Three groups were analyzed: patients treated with preoperative PD-1 inhibiting ICI as monotherapy or in concert with a VEGF tyrosine kinase inhibitor, as clinically indicated for downstaging (preoperative ICI); patients without preoperative ICI with clinical recurrence (treatment-naïve with recurrence); and patients without preoperative ICI without clinical recurrence (treatment-naïve without recurrence). No patients in this cohort received adjuvant therapy.

### 2.2. Gene and Protein Expression Analysis

Tumor microarrays (TMAs) were constructed from multiple 1 mm-diameter cores from formalin-fixed and paraffin-embedded tumor samples for a total of 41 cores (2–3 per tumor). Within each TMA, 400–660 µm-diameter regions of interest (ROIs) were analyzed using Nanostring GeoMx Digital Spatial Profiler (DSP) protein analysis (an 87-protein oncology-specific panel) and Nanostring GeoMx DSP Human Whole-Transcriptome Atlas RNA analysis (>18,000 genes) [6,7]. Selected ROIs for analysis were concentrated areas of tumor cells without significant intratumoral necrosis, major vasculature, or processing-related artifact. The ROIs were processed and analyzed per manufacturer protocols [8].

Reagents consisted of a suspension of DSP oligonucleotide barcodes attached via a photocleavable linker to either target-specific antibodies or complementary nucleotide sequences for respective protein and RNA analyses. TMAs were then stained with imaging and assay probes, using respective protein or RNA reagents. Barcodes from the ROIs were released via ultraviolet light and quantified via the nCounter analysis system [9]. For protein analysis, raw counts were normalized using the geometric mean of housekeeping proteins and immunoglobulin controls. For RNA analysis, proprietary positive and negative controls were introduced to ensure quality control, and then Q3 normalization was performed, resulting in similar gene expression ranges for samples.

Normalized counts were analyzed individually for heterogeneity and averaged on a per-patient level. DSP protein data were filtered for 15 proteins relevant to immune cell phenotype and function. After quality control, DSP RNA data included 16,173 genes. RNA data were normalized using *voom* (version 3.66.0) to estimate the mean–variance relationship and generate precision weights for subsequent differential expression analysis with *limma *(version 3.66.0) [10,11]. Enrichment analysis using the *Reactome* 2024 gene set (version v91) was applied to clinically relevant sets of differentially expressed genes [12]. *xCell* immune cell deconvolution was applied to normalized RNA data to estimate relative immune cell content between samples [13].

Heatmaps were generated for the protein, RNA, and *xCell *(version 2017) data using *pheatmap* (version 1.0.13) and then stratified by the preoperative ICI, treatment-naïve without recurrence, and treatment-naïve with recurrence groups [14]. Jittered plots were generated for protein, RNA, and *xCell* results with false discovery rate (FDR)-adjusted non-parametric *p*-values to determine statistical significance between treatment groups. Spearman’s correlations were determined for matched pairs of genes and protein products.

### 2.3. External Validation and Generation of Gene Score for Prognostication

We validated our differential expression analysis using NCT02210117 data [15]. In that trial, patients with metastatic ccRCC without prior exposure to anti-CTLA-4, anti-PD1, or bevacizumab agents were randomized to various ICI-containing regimens before undergoing cytoreductive nephrectomy or biopsy. The primary endpoint of that study was safety; secondary endpoints included best overall response, progression-free survival, overall survival (OS), and correlative immunologic changes.

To identify genes that were (1) associated with favorable clinical outcomes and (2) modifiable with ICI, differentially expressed genes associated with nonrecurrence and ICI exposure from our initial experiment were utilized as an initial set. These genes were matched with those of patients in the trial who had RNA analysis both from pretreatment and post-ICI tissue, which reported 737 genes from the Nanostring Pan-cancer Immune Profiling assay. The resulting matched genes were then further filtered by selecting genes with significantly increased expression after ICI exposure, as well as objective treatment response per RECIST criteria [16].

To determine which cell types expressed these genes, the gene set after validation and refinement with NCT02210117 data (*GZMA*, *GZMK*, *IL7R*, and *ITGAL*) was assessed using a meta-analysis of scRNA-seq data derived from 14 experiments on ccRCC tumors, facilitated by *UncoVer* [17]. Uniform manifold approximation and projection (UMAP) was applied to scRNA-seq data; then, broadly generalizable clusters of immune, stromal, and tumoral cells were identified using canonical gene markers. Expression levels of each gene in our final set were plotted to determine cell types expressing these genes.

The final gene set after validation/refinement with NCT02210117 data was used to create a gene score using the geometric means of gene expression levels. This score was applied to treatment-naïve stage III patients in The Cancer Genome Atlas clear cell RCC cohort (TCGA KIRC, *n* = 123) to determine associations with OS. The TCGA was accessed using the *TCGAbiolinks* package. Patients were stratified by the median gene score into high and low scores; then, Kaplan–Meier survival curves were generated.

### 2.4. Statistical Analysis

Computational and statistical analyses were conducted using R version 4.4.2 (R Foundation for Statistical Computing). Statistical significance was determined using non-parametric FDR-adjusted *p* < 0.05. Kruskal–Wallis rank sum testing was utilized to test statistical differences between the three groups. When appropriate, Wilcoxon signed-rank or rank sum testing was used to test statistical differences when two groups were present. Survival analysis was conducted using the *survival* (version 3.8-6) and *survminer *(version 0.5.1) packages.

## 3. Results

Nineteen patients with stage III ccRCC were identified; basic characteristics of the cohort are described in Table S1. Four patients received preoperative PD-1 ICI therapy, of whom three had clinical recurrence. Among the fifteen patients without preoperative ICI, eight experienced a recurrence, and seven were disease-free at ≥48 months follow-up.

**Figure 1:**
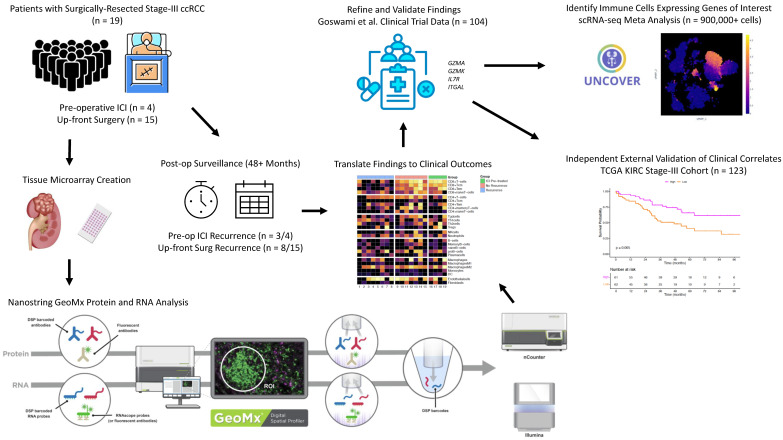
Schematic of study design and workflow [15].

**Figure 2:**
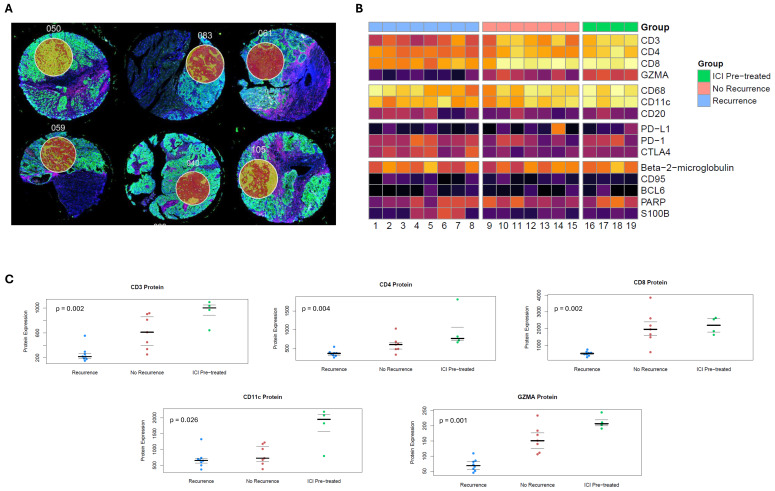
(**A**) Examples of region of interest (ROI) selection using Nanostring GeoMx Digital Spatial Profiler post-staining and pre-cleaving, which enables precise sampling of concentrated tumoral regions. Areas of necrosis, major vasculature, tissue artifact, and normal adjacent kidney were avoided. (**B**) Heatmap for expression of 15 proteins relevant to immune cell function for patients who were treatment-naïve with recurrence (blue), treatment-naïve without recurrence (pink), or with preoperative ICI exposure (green). (**C**) Jittered plots for protein expression where protein expression significantly differed between the three groups, which included CD3, CD4, CD8, CD11c, and GZMA. The bold line represents the median, and thin lines represent the interquartile range.

Among 19 specimens, 41 cores were obtained to create TMAs (2–3 cores per specimen); 41 RNA (1 per core) and 54 protein ROIs (1–3 per core) were selected for analysis. Our workflow is depicted in Figure 1. Representative examples of how DSP was leveraged to select tumor ROIs are shown in Figure 2A.

### 3.1. Protein Marker Analysis

Protein expression of CD3, CD4, CD8, GZMA, and CD11c significantly differed between the three groups (Figure 2B). Higher expression of these proteins was seen in the preoperative ICI and treatment-naïve without recurrence groups than in the treatment-naïve with recurrence group (Figure 2C). Notably, the immune checkpoints PD-1, PD-L1, and CTLA-4 did not significantly differ.

### 3.2. Gene Expression Analysis

**Figure 3:**
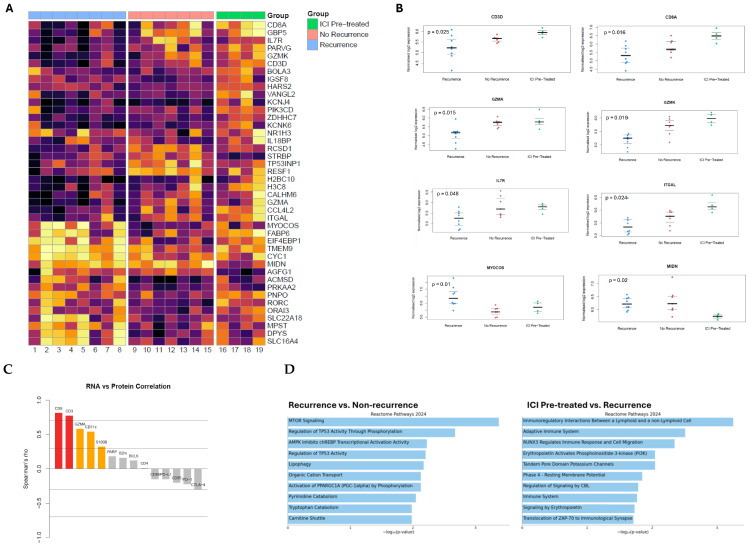
(**A**) Heatmap for RNA expression of genes that were most variably expressed between patients who were treatment-naïve with recurrence (blue), treatment-naïve without recurrence (pink), and with preoperative ICI exposure (green). (**B**) Normalized log2 RNA expression for selected genes that significantly differed across the three groups. (**C**) Plot of Spearman’s correlation coefficients for RNA and corresponding protein expression. (**D**) Reactome 2024 gene expression pathway comparing patients who were treatment-naïve with recurrence versus treatment-naïve without recurrence, as well as patients with preoperative ICI exposure versus treatment-naïve with recurrence.

RNA data was filtered for the most variably expressed genes between groups (Figure 3A). Normalized RNA expression significantly differed for *CD3D*, *CD8A*, *GZMA*, *GZMK*, *ITGAL*, *IL7R*, *MIDN*, *TMEM9*, and *MYOCOS* (Figure 3B), as well as several other genes reported in Table S2.

Spearman’s correlations were determined between paired RNA and protein product expression in matched tissues. There were strong correlations for CD8 (r = 0.81, *p* < 0.001) and CD3 (r = 0.77, *p* < 0.001); moderate correlations for GZMA (r = 0.58, *p* = 0.01), CD11c (r = 0.54, *p* = 0.02), and S100B (r = 0.32, *p* = 0.18); and poor correlations for the remainder of the RNA–protein pairs (Figure 3C). Notably, immune checkpoints demonstrated weak negative correlations between gene expression and protein product (PD-L1: r = −0.15, *p* = 0.53; PD-1: r = −0.22, *p* = 0.36; CTLA-4: r = −0.29, *p* = 0.21).

Reactome 2024 gene expression pathway analysis was conducted using the between-groups differential expression analysis (Figure 3D). Tumors from treatment-naïve patients with recurrence had “MTOR Signaling” (*p* < 0.001) and “Regulation of TP53 Activity Through Phosphorylation” (*p* = 0.002) pathway enrichment, among others. Preoperative ICI tumors had enrichment of “Immunoregulatory Interactions Between a Lymphoid and a non-Lymphoid Cell” (*p* < 0.001) and “Adaptive Immune System” (*p* = 0.003) pathways, among other ones.

**Figure 4:**
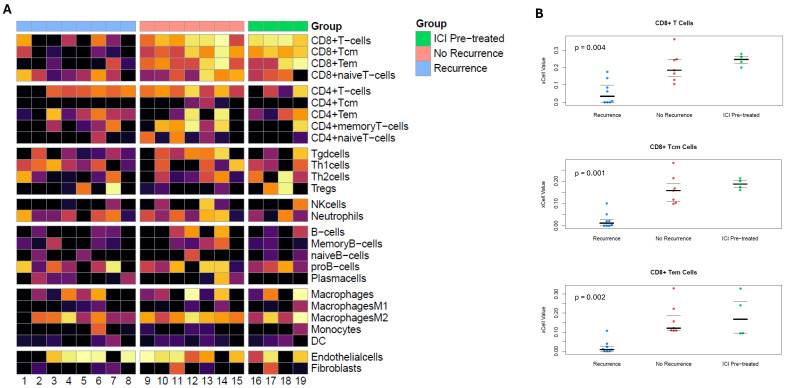
(**A**) Heatmaps for relative prevalence of immune cell subtypes based on *xCell* immune cell deconvolution applied to RNA expression data. (**B**) Jittered plots for relative prevalence of CD8+ T cells, CD8+ T_cm_ cells, and CD8+ T_em_ cells that show significant differences across the three groups (preoperative ICI, treatment-naïve without recurrence, and treatment-naïve with recurrence).

Gene expression data from the RNA analysis was utilized for immune cell deconvolution analysis using the *xCell* algorithm [13]. Gene expression signatures significantly differed between groups for CD8+ T-cell, CD8+ Tcm-cell, and CD8+ Tem-cell subtypes (Figure 4A). These immune cell subtype signatures were significantly higher in the preoperative ICI and treatment-naïve without recurrence groups and lower in treatment-naïve patients with recurrence (Figure 4B). Although the CD11c protein, a marker for dendritic cells, had significantly higher expression in the ICI pretreatment group, there were no significant differences in the prevalence of dendritic cells noted with *xCell*.

### 3.3. Validation of Gene Expression Results

**Figure 5:**
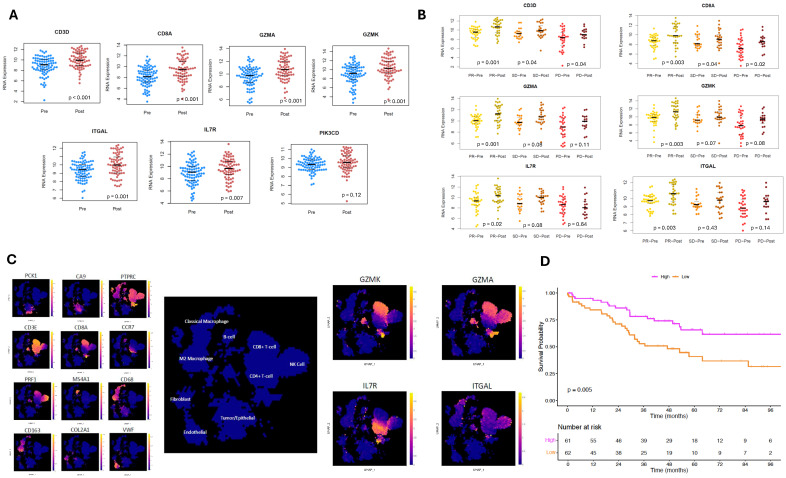
(**A**) Jittered plots of the 7 genes with significant differential expression in our study that overlapped with RNA-seq data in the NCT02210117 clinical trial, based on pre- (blue) and post-ICI (red) exposure. (**B**) Jittered plots stratified by treatment response (partial response [PR], stable disease [SD], and progression of disease [PD]) for the 6 genes that showed significant pre- versus post-ICI differences. (**C**) Meta-analysis of single-cell RNA-seq data from ccRCC tumor tissue using the *UncoVer* platform; cell identities in the UMAP were determined using canonical gene markers, and then, expression of *GZMA*, *GZMK*, *IL7R*, and *ITGAL* was mapped to these clusters. (**D**). Kaplan–Meier curves for overall survival based on clinical stage III ccRCC patients from the TCGA KIRC cohort stratified by high (magenta) and low (orange) gene scores based on expression levels of *GZMA*, *GZMK*, *IL7R*, and *ITGAL*.

We ultimately matched seven genes from our gene expression analysis associated with the preoperative ICI and treatment-naïve without recurrence groups to RNA data from the NCT02210117 trial (Figure 5A). Further limiting this set of genes to only those that were significantly increased after ICI exposure yielded six genes (Figure 5B). Finally, limiting this set to genes that had significantly increased expression in patients with objective treatment response yielded four genes: *GZMK*, *GZMA*, *ITGAL*, and *IL7R*.

### 3.4. External Analysis of Immune Cell Phenotype

On scRNA-seq meta-analysis of ccRCC tumors, there were distinct expression patterns based on different cell types noted (Figure 5C). *GZMK* is primarily expressed in CD8+ T-cells, *GZMA* in CD8+ T-cells and NK cells, *IL7R* in CD4+ T-cells, and *ITGAL* in CD8+ T-cells, NK cells, and classical macrophages.

### 3.5. Stratification of Survival Outcomes Based on Gene Scores

The final gene set (*GZMK*, *GZMA*, *IL7R*, and *ITGAL*) was adapted into a gene score and then applied to an independent cohort of treatment-naïve patients with stage III ccRCC from the TCGA-KIRC cohort (*n* = 123). Kaplan–Meier estimates demonstrated significantly improved OS for patients with higher gene scores (log-rank *p* = 0.005, Figure 5D).

## 4. Discussion

Multimodal DSP of stage III ccRCC tumors found that protein and RNA expression indicative of increased CD8+ Tcm and Tem cells was associated with a durable cancer-free state postoperatively. Interestingly, patients with preoperative ICI had similarly favorable findings but without corresponding improved outcomes; three out of four of these patients experienced recurrence.

We further investigated, validated, and refined our findings using several independent external cohorts. In doing so, we identified *GZMK*, *GZMA*, *IL7R*, and *ITGAL* as genes that are potentially both modifiable with ICI therapy and associated with favorable clinical outcomes. As such, patients whose tumors have low baseline expression of these genes may benefit most from ICI around the time of surgery—a hypothesis that we intend to directly test in future experiments.

Dysfunction in CD8+ T cells has been shown to be associated with more advanced ccRCCs in prior single-cell transcriptomic data [18]. This T-cell exhaustion may contribute to non-durable responses to immune checkpoint inhibition. Thus, identifying biomarkers that can predict potential T-cell activity with immune checkpoint inhibition in this high-risk population is key. More traditional biomarkers, such as PD-L1 positivity and tumor mutational burden (TMB), have shown a limited or mixed ability in predicting response to immune checkpoint inhibitors [19]. Responses to immune checkpoint inhibition have been demonstrated regardless of PD-L1 expression levels, and RCCs are typically associated with low TMB.

Research assessing biomarkers for response is ongoing along multiple fronts, including somatic alterations, transcriptional signatures, and circulating DNA, among other modalities [20]. Within the context of metastatic ccRCC, unsupervised transcriptomic analysis has been used to identify ccRCC subtypes based on a 35-gene signature to predict response to tyrosine kinase inhibitors [21], which in turn has been prospectively evaluated to select different treatment regimens in this population [22].

In our study, four genes that were validated in external cohorts emerged as potential predictors for response to ICI. We reviewed the literature regarding these genes in relation to RCC and malignancy more broadly.

*GZMK* encodes serine protease granzyme K, which is normally expressed by CD8+ T cells, NK cells, and innate-like T cells [23]. Granzyme K facilitates apoptosis of target cells through single-stranded DNA nicks and damage to mitochondrial membranes [24,25]. In the context of RCC, clinical trials assessing PD-1 ICI in patients with advanced RCC found that CD8+ T cells expressing *GZMK* were significantly more prevalent in responders [26,27]. Outside of RCC, analysis of TCGA cohorts has also noted improved OS among breast cancer patients with higher *GZMK* expression, and higher levels of *GZMK* have been noted in melanomas responsive to nivolumab, a disease setting noted for its immunogenicity [28].

*GZMA* encodes granzyme A and is also a serine protease with tryptase activity prevalent in CD8+ T cells and NK cells [29]. Within the context of RCC, tumors with higher levels of NK cells had higher granzyme A expression, although this was not correlated with oncologic outcomes [30]. *GZMA* expression was also associated with higher lymphocytic infiltration and T cell gene expression in patients with localized RCC [31]. Regarding prognosis, *GZMA* expression was not associated with OS among patients with ccRCC, though that particular cohort was primarily composed of stage I-II tumors [32].

*IL7R* encodes the alpha chain of IL-7R, a cell-surface receptor that promotes lymphocyte development and cell survival through the JAK/STAT signaling pathway [33]. In a mouse model with a chronic viral infection, PD-1 blockade led to increased *IL7R* expression and IL-7 signaling, which was associated with transient reinvigoration of exhausted CD8+ T cells [34]. The literature for *IL7R* within RCC is sparse, with increased proliferation of tumor-infiltrating lymphocytes having been observed after IL-7 treatment [35]. In our meta-analysis of single-cell transcriptomic data in ccRCC tumors, *IL7R* was predominantly expressed in CD4+ T cells. Interestingly, in a separate study of patients with advanced or metastatic RCC, high expression of CD4+ T cells was associated with a lower likelihood of ICI response [36]. It must be noted, however, that this does not account for the various subtypes of CD4+ T cells, such as regulatory T cells that have been more often associated with poor treatment response. In that same study, the presence of CD68+ macrophages that infiltrated into tumors was also a significant predictor of ICI response [36]; our study did not show significant differences in macrophage prevalence across the three groups.

*ITGAL* codes for the integrin alpha L chain, which is a subunit of the lymphocyte function-associated antigen-1 (LFA-1) critical for leukocyte migration. RCCs overexpress *ITGAL* compared to normal tissue; additionally, greater expression is associated with higher tumor grade. That same study also found that within a 10-gene expression signature, greater *ITGAL* expression predicted poorer survival using data from TCGA [37]. The study did not stratify *ITGAL* expression within stages in relation to survival, though. Outside of RCC, lower ITGAL expression in non-small-cell lung cancer is predictive of poorer prognosis, with higher immune infiltration in malignant tissues [38].

Of note, the assumption is often made that the expression of a gene and its downstream protein are highly correlated. However, our data did not demonstrate this. Of fourteen matched gene–protein pairings, only two strongly correlated (CD8 and CD3), and three moderately correlated (GZMA, CD11c, and S100B). Notably, the immune checkpoint proteins PD-L1, PD-1, and CTLA-4 all demonstrated weak negative correlations, which have previously been noted [39,40,41,42]. These data underscore the point that protein and RNA expression levels are not interchangeable, especially for immune checkpoint proteins. Although our study was not designed to assess the reasons for these discrepancies, regulatory mechanisms at different levels could account for this. Translational efficiency of mRNA transcripts may vary based on transcript modifications and the presence of RNA silencing. At the protein level, the stability of these products may be variable and affected by post-translational modifications. Together, this does underscore the importance of continuing to assess the correlation of RNA and protein expression levels in future studies.

### Limitations

Our cohort was small (*n* = 19 patients), which limited power; we did however validate these findings using larger independent studies. This experiment was designed to favor a deep and comprehensive molecular analysis of fewer specimens, as opposed to a superficial analysis of many specimens. Future studies validating these findings will be more targeted and feature a larger sample size.

Our validation set from the NCT02210117 trial was limited by its recruitment of patients with metastatic ccRCC, unlike patients in KEYNOTE-564 and PROSPER. The patients enrolled were naïve to various ICIs and bevacizumab, though, thereby reducing the risk of confounding from heavy preoperative exposure to these agents. Transcriptional and proteomic data from large clinical trials that precisely match our patient cohort would be valuable in the further validation of our findings, but these are lacking.

Additionally, the lack of single-cell resolution limits immune cell phenotyping to relatively broad subtypes, thereby limiting the analysis of granular subtypes of exhausted T-cells and anti-inflammatory macrophages, whose importance has been previously described [18,43,44]. Interestingly, expression levels in the nonrecurrence group were lowest for *LAG3* and *HAVCR2*, which are markers that have been associated with T-cell exhaustion [45,46]. However, the differences in RNA expression across the groups did not meet statistical significance.

Lack of spatially resolved protein or RNA expression data at the single-cell level also did not allow for the description of CD8+ clustering patterns to better describe immune infiltration [40] or the presence of tertiary lymphoid structures that have been shown to play an important role in anti-tumor responses [47]. Ultimately, the described findings absolutely require further validation before practical application to clinical practice.

## 5. Conclusions

Our analysis found that stage III ccRCC patients with preoperative ICI had increased intratumoral gene expression indicative of CD8+ T-cell effector and central-memory phenotypes. Similar findings were seen in untreated patients without recurrence. Despite this favorable immune composition, though, our ICI-pretreated patients recurred at similar rates to untreated patients. Overall, receipt of preoperative ICI appeared to favorably alter the tumor immune microenvironment. However, this did not result in convincingly better clinical outcomes.

Further refinement using NCT02210117 trial data suggested that increased gene expression of *GZMK*, *GZMA*, *ITGAL*, and *IL7R* is inducible with ICI and associated with favorable outcomes. Patients whose tumors have low initial expression of these genes may derive the most benefit from ICI around the time of surgery. Once published, data from the PROSPER and KEYNOTE-564 trials will be critical to test this hypothesis.

## Data Availability

The data that support this study’s findings are available from the authors upon reasonable request.

## Supplementary Materials

The following supporting information can be downloaded at: https://www.mdpi.com/article/10.3390/cancers18020312/s1, Table S1: baseline characteristics of patients included in the study; Table S2: genes with significantly variable expression based on study group, as well as genes matched to data from the NCT02210117 trial based on upregulation with immune checkpoint blockade and objective response.
